# Dynamic Responses of Ascorbate Pool and Metabolism in Lettuce to Light Intensity at Night Time under Continuous Light Provided by Red and Blue LEDs

**DOI:** 10.3390/plants10020214

**Published:** 2021-01-23

**Authors:** Yuan Wen, Lingyan Zha, Wenke Liu

**Affiliations:** 1 Institute of Environment and Sustainable Development in Agriculture, Chinese Academy of Agricultural Sciences, Beijing 100081, China; wenyuan@caas.cn (Y.W.); zhaly@sjtu.edu.cn (L.Z.); 2 Key Lab of Energy Conservation and Waste Management of Agricultural Structures, Ministry of Agriculture and Rural Affairs, Beijing 100081, China

**Keywords:** continuous light, dark period light intensity, ascorbate, ascorbate-glutathione cycle, enzyme activity

## Abstract

To understand the dynamic changes of hydroponic lettuce growth, ascorbate (AsA) pool and metabolism under two different dark period light intensities (LL, 20 μmol·m^−2^·s^−1^; CL, 200 μmol·m^−2^·s^−1^) of continuous light and normal light (NL, 0 μmol·m^−2^·s^−1^) provided by red (R) and blue (B) LEDs, the chlorophyll fluorescence parameters, ascorbate pool size, AsA metabolism-related enzyme activities, and H_2_O_2_ contents of lettuce were measured at 0, 8, 16, 24, 32, 40, 48, 56, 64, and 72 h after light treatment and the lettuce growth parameters were measured on the 9th day after light treatment. The results showed that compared with the NL, CL treatment for 9 days significantly increased the biomass, dry matter content, and specific leaf weight of lettuce, but had no significant effect on the leaf area and root-to-shoot ratio; LL had no significant effect on lettuce biomass, but it would reduce the root-shoot ratio. Compared with the NL, the AsA content of CL increased significantly within 8 h after light treatment (at the end of first dark period), and then maintained at a relatively stable level with a slight increase; there was no significant difference in AsA contents between NL and LL showing the same circadian rhythm characteristics. Overall, the activities of L-galactono-1,4-lactone dehydrogenase (GalLDH), ascorbate peroxidase(APX), monodehydroascorbate reductase (MDHAR), and glutathione reductase (GR) under CL were the highest among the three treatments, and the differences with the other two treatments reached significant levels at several time points; there was almost no significant difference in the activities of GalLDH, APX, MDHAR, and GR between NL and LL; there was no significant difference in the activities of dehydroascorbate reductase (DHAR) under different treatments. Compared with the NL, CL caused a sharp decrease of PSⅡ maximal photochemical efficiency (Fv/Fm) in lettuce within 0–8 h after treatment, which then stabilized at a relatively stable level; the Fv/Fm value under the LL was almost the same as the NL. Except for 32 h, the H_2_O_2_ content of lettuce under CL was the highest among the three treatments during the entire experimental period, and was significantly higher than that of NL at several time points; the H_2_O_2_ content of LL was almost the same as NL. In summary, lettuce biomass, AsA contents, AsA metabolism-related enzyme activities, chlorophyll fluorescence parameters, and H_2_O_2_ contents were regulated by the dark period light intensities of continuous light rather than continuous light signals.

## 1. Introduction

The plant factory based on hydroponic technology is the most advanced form of modern facility gardening, which can significantly improve the efficiency of vegetable production. Leafy vegetables have become one of the main vegetable types for hydroponics in plant factories due to their short cultivation time, short plant shape, and high market demand. Compared with natural light conditions, the overall light level of greenhouses and plant factories is relatively weak. The lower light level is one of the main reasons for the low ascorbate (AsA) content of hydroponic leaf vegetables in plant factories [1]. As a very important antioxidant substance, AsA plays an extremely important role in maintaining human health and the normal growth of plants [2,3,4,5]. The human body needs to maintain the AsA content above the critical level, but humans lack the L-gulono-γ-lactone oxidase that catalyzes the last step of AsA biosynthesis [4], and related genes have accumulated harmful mutations during the evolution process [6,7,8]. Therefore, humans cannot synthesize AsA by themselves and can only rely on the ingestion of exogenous AsA. In addition, AsA is highly water-soluble and cannot be stored in the human body. It must be ingested continuously to ensure human health. Vegetables are one of the most important sources of AsA in the human diet. Increasing the AsA content in vegetables is of great significance for maintaining human health [9,10]. Besides, AsA has the functions of resisting oxidative stress and photooxidation [11], acting as a coenzyme [12], and regulating growth and development in plants [13,14,15].

The new semiconductor light source LED has excellent photoelectric characteristics such as high light efficiency, energy saving, small size, long use time, and environmental protection, and is currently an ideal light source for facility gardening and artificial light cultivation [16,17]. People can create an artificial light environment with adjustable light intensity, light quality, and photoperiod according to the needs of plants for light. In the natural environment, there are regular 24-h day and night changes in environmental conditions such as light and temperature. In order to adapt to the external environment, many physiological activities of plants have also formed a circadian rhythm that is synchronized with the external environment. The biological rhythm cycle and the various physiological changes of plants can be regulated by environmental cycle rhythms [18]. The content of AsA and the activities of some enzymes in its metabolism have been confirmed by previous studies to have circadian rhythm characteristics [19]. However, there is no research on the characteristics of AsA rhythm changes under continuous light.

Strictly continuous light means that the light intensity and light quality are constant [20], but from the perspective of light signal, as long as the plant is continuously provided with light for more than 24 h, it can be regarded as continuous light. As we know, light is an environmental vital factor to the accumulation of AsA in plants [21], by providing photosynthetic energy and environmental signal. The regulation of AsA by light depends on the electron transfer caused by light and the reactive oxygen species (ROS) produced in the chloroplast under light, and light intensity is the decisive light environmental factor that affects photosynthetic electron transfer and ROS. The role of AsA in resisting photooxidative stress also indicates that light intensity can regulate AsA content. A large number of studies have shown that within a certain range of light intensity, the greater the light intensity, the higher the AsA content in plants [22,23,24]. For instance, the AsA content in spinach leaves under 200 μmol·m^−2^·s^−1^ light intensity was significantly lower than that in spinach leaves grown under 800 μmol·m^−2^·s^−1^ [25] and the AsA content in lettuce leaves grown under 100 μmol·m^−2^·s^−1^ light was significantly lower than that of lettuce leaves grown under 300 μmol·m^−2^·s^−1^ [26]. The dependence of AsA content in plants on light intensity indicated that light energy participated in the process of light regulating AsA accumulation [27,28,29,30]. However, the current research has not clarified whether the influence of light on the AsA content includes the signal effect. Studies have shown that the activation of blue (B) light receptor and red (R) light receptor phytochrome is involved in the synthesis of AsA [31,32]. In addition, there is a correlation between GSH in the ascorbate-glutathione cycle (AsA-GSH cycle) and photosensitive signal [33]. These studies indirectly prove that light signal may have a regulatory effect on AsA metabolism. Whether the promotion effect of continuous light on AsA is related to continuous light signal is still uncertain.

Under continuous light condition, low light intensity in the dark period was used to study the effects of light signal on plant biological rhythm [34]. In this study, the normal photoperiod was used as a control (16/8 h) to monitor the diurnal changes in the AsA content and metabolic-related enzyme activities of lettuce in 72 h under continuous light. At the same time, the continuous light processing of low light in the dark period was set to explore whether the light signal played a role in the continuous light to promote the accumulation of AsA. This study aimed to explore the relationship between the physiological metabolism and biological rhythms of AsA and the regular changes of environmental cycles, and to provide new ideas for in-depth understanding of the adaptive mechanism of plants to the environment.

## 2. Materials and Methods

### 2.1. Plant Materials and Growth Condition

The experiment was carried out in an experimental plant factory of the Institute of Environment and Sustainable Development in Agriculture of the Chinese Academy of Agricultural Sciences. The “Italian bolting-resistant” lettuce (*Lactuca sativa* L. cv. ‘Yidali’) was selected as the experimental material. When raising seedlings, lettuce seeds were sowed in a soaked sponge seedling block (2.5 × 2.5 × 2.5 cm) and placed in a dark environment. After the seeds germinated until the seed coat fell off, the lettuce seedlings were transferred to the white LED light for seedling raising. The light intensity and photoperiod of the seedlings were 200 μmol·m^−2^·s^−1^ and 16/8 h (light/dark), respectively. During the seedling raising period, the nutrient solution was added to the seedling tray every two days. After the seedlings were grown to two leaves and one heart (two weeks), the seedlings were transplanted to the nutrient solution circulation cultivation system for cultivation and light treatment. The light source installed in the cultivation system was red and blue LED light panels with adjustable light quality ratio. The nutrient solution formula used for the growth of lettuce was Hoagland nutrient solution, circulating for 30 min every day after transplanting. During the experiment, the day and night temperature and relative humidity in the plant factory were maintained at 23 ± 1/20 ± 1 °C and 50–60%, respectively. The CO_2_ concentration was about 400 ppm.

### 2.2. Light Treatments and Sampling Time

In order to adapt the lettuce seedlings to the nutrient solution circulation cultivation system, the seedlings were transplanted and then grown for 3 d under uniform light conditions (3R:1B, 200 μmol·m^−2^·s^−1^, 16/8 h). Then, the lettuce seedlings were randomly divided into three groups (78 plants per group) and treated with three different light intensities in the dark period. The three light treatments were normal light (NL, 0 μmol·m^−2^·s^−1^), low light (LL, 20 μmol·m^−2^·s^−1^), and continuous light (CL, 200 μmol·m^−2^·s^−1^) (Table 1). The light quality and light intensities of light period of all treatments were 3R:1B and 200 μmol·m^−2^·s^−1^, respectively. Samples were taken at 0, 8, 16, 24, 32, 40, 48, 56, 64, and 72 h of each treatment, and five plants were randomly selected as five biological replicates for each treatment. After sampling, we removed the old and new leaves of each plant, selected the middle 4–5 leaves and removed the veins, put them in a foil paper bag and immediately placed them in liquid nitrogen, and transferred them to −80 °C refrigerator after freezing for the determination of various physiological indicators.

### 2.3. Measurement Methods

#### 2.3.1. Measurement of Growth Parameters

At the end of the light period on the 9th day, five plants of each treatment were taken as five replicates to determine the growth parameters. The above-ground and underground parts from the base of lettuces were separated, and an analytical balance was used to weigh the dry and fresh weights of them, respectively. After weighing, the leaf veins were removed, and the leaf area and leaf fresh weight were measured by a leaf area meter and a balance, respectively. Specific leaf weight was calculated by leaf area and leaf fresh weight. Finally, the whole plant was dried in an oven at 80 °C to a constant weight and the dry weight of the plant was weighed.

#### 2.3.2. Measurement of Chlorophyll Fluorescence Parameters

MINI-PAM (Germany, WALZ) was used to measure the dark adaptation maximum (Fm) and minimum (Fo) fluorescence at 0, 8, 16, 24, 32, 40, 48, 56, 64, and 72 h of each light treatment. PSⅡ maximal photochemical efficiency (Fv/Fm) was calculated according to the formula Fv/Fm = (Fm − Fo)/Fm.

#### 2.3.3. Measurement of AsA Content

AsA content was measured by the method of GILLESPIE and AINSWORTH [35]. Accurately 0.1 g frozen fresh sample was weighed into a 2 mL centrifuge tube, and a high-throughput tissue grinder (Ningbo Xinzhi, Scientz-48) was used to grind the sample to powder under low temperature condition maintained by liquid nitrogen. After grinding, 1 mL of precooled 6% (*w/v*) trichloroacetic acid was added to the centrifuge tube, vortexed to mix immediately, and ultrasonically extracted in ice water for 12 min, and then centrifuged at 13,000× *g* for 15 min at 4 °C. To determine the total AsA (T-AsA), 200 μL of the supernatant was taken, 100 μL of 75 mM phosphate buffer solution (PBS) and 100 μL of 10 mM DTT (to reduce DHA to AsA) was added and reacted at room temperature for 10 min, then 100 μL 0.5% NEM (to stop the reaction), 500 μL 10% TCA, 400 μL 43% H_3_PO_4_, 400 μL 4% α-α-bipyridine, and 200 μL 3% FeCl_3_ were added and mixed quickly, then reacted in a 37 °C water for 1 h. After the reaction, the absorbance was measured at 525 nm. When measuring the reduced AsA (AsA), 200 μL H_2_O was used instead of DTT and NEM, and the remaining steps were the same as those of T-AsA. The oxidation state AsA (DHA) content was the difference between T-AsA and AsA.

#### 2.3.4. Measurement of AsA Metabolism-Related Enzyme Activity

The activity of L-galactono-1,4-lactone dehydrogenase (GalLDH) was determined according to the method of CARLOS and EDUARDO [31]. A total of 0.3 g frozen sample was taken and mixed with 2 mL prechilled 100 mM PBS (PH 7.4, containing 0.4 M sucrose, 10% (*v/v*) glycerol, 1 mM EDTA, 0.3% (*v/v*) mercaptoethanol, 1% (*w/v*) polyvinylpyrrolidone (PVP)), and then the mixture was ground into a homogenate. The homogenate was centrifuged at 500× *g* for 10 min at 4 °C, and the supernatant was centrifuged at 12,000× *g* for 20 min at 4 °C. The precipitate was resuspended in 0.2 mL 100 mM PBS (PH 7.4, containing 5 mM GSH sucrose and 10% (*v/v*) glycerol) to obtain the enzyme extract. In the measurement, 1 mL of the reaction solution contained 50 mM PBS (pH 7.8), 1.05 mg/mL cytochrome c (Cyt c), 5.6 mM L-galactono-1,4-lactone, and 0.1 mL enzyme extract. It was defined that the amount of enzyme required to oxidize 1 nmol L-galactono-1,4-lactone or generate 2 nmol of reduced Cyt c in 1 min was an enzyme activity unit, and the molar coefficient used in calculating the enzyme activity was 17.3 mM/cm.

Ascorbate peroxidase (APX) activity was determined with reference to NAKANO and ASADA method [36]. After 0.1 g frozen fresh sample was ground in liquid nitrogen, 1 mL of prechilled 50 mM PBS was added into it and then the mixture was vortexed immediately. The mixture was ultrasonically extracted in ice water for 12 min, and then centrifuged at 13,000× *g* for 15 min at 4 °C. The supernatant was used to determine APX activity. During the determination, 2.6 mL 50 mM PBS (containing 0.1 mM EDTA and 0.5 mM AsA) and 0.1 mL enzyme extract was added to the cuvette, and finally 0.3 mL 2 mM H_2_O_2_ was added to start the reaction, and mixed immediately. The absorbance value was read within 180 s at 290 nm, every 20 s.

The activities of dehydroascorbate reductase (DHAR), monodehydroascorbate reductase (MDHAR), and glutathione reductase (GR) were measured with reference to the method of MA and CHENG [37]. After 0.1 g of frozen fresh sample was ground in liquid nitrogen, 1 mL of prechilled 50 mM PBS (PH 7, containing 1 mM EDTA, 1 mM DTT, 2% (*w/v*) PVP, 0.1% (*v/v*) Triton X and 0.2% (*v/v*) mercaptoethanol) was added and vortexed immediately. The mixture was ultrasonically extracted in ice water for 12 min, and then centrifuged at 13,000× *g* for 15 min at 4 °C. The supernatant was used to determine DHAR, MDHAR, and GR activities. When measuring DHAR activity, the 3 mL reaction system contained 2.8 mL 100 mM Hepes-KOH (pH 7.0, containing 1 mM EDTA and 2.5 mM GSH), 0.1 mL 6 mM DHA, and 0.1 mL enzyme extract. The amount of enzyme required to oxidize 1 mM NADH per minute was defined as one unit of enzyme activity. When measuring MDHAR activity, a 3 mL reaction system contained 2.7 mL 50 mM Hepes-KOH (pH 7.6, containing 0.5 mM AsA), 0.1 mL 3 mM NADH, 0.1 mL 5 U/mL AO, and 0.1 mL enzyme extract. When measuring GR activity, a 3 mL reaction system contained 2.7 mL 100 mM Tris-HCl (pH 8.0, including 1 mM EDTA), 0.1 mL 30 mM GSSG, 0.1 mL 6 mM NADPH, and 0.1 mL enzyme extract.

#### 2.3.5. Measurement of H_2_O_2_ Content

The H_2_O_2_ content was determined by the method of Brennan and Frenkel [38]. A total of 0.1 g frozen fresh sample was weighed and ground into powder in liquid nitrogen. Then, 1 mL precooled acetone was used to extract H_2_O_2_. The homogenate was centrifuged at 10,000× *g* for 20 min at 4 °C. After centrifugation, we drew the supernatant, added 0.2 mL concentrated ammonia and 0.1 mL 10% (*v/v*) titanium tetrachloride-hydrochloric acid solution, mixed them immediately, and then centrifuged at 4000× *g* for 10 min at 25 °C. After discarding the supernatant, 1 mL acetone was added to wash off the pigment in the pellet. The mixture was then centrifuged again (25 °C, 4000× *g*, 10 min). We discarded the supernatant, dissolved the precipitate with 1 mL 2 M H_2_SO_4_, and measured the absorbance of the solution at 412 nm.

## 3. Results

### 3.1. Effect of Light Intensities at Night Time on Growth of Lettuce under Red and Blue Continuous Light

As shown in the Table 2, after 9 d treatment with different dark period light intensities, the above-ground fresh weight, above-ground dry weight, root dry weight, specific leaf weight, and dry matter content of lettuce under CL were significantly higher than the other two treatments. Compared with the NL, the above-ground biomass of lettuce under LL increased slightly, but the root biomass decreased slightly. Therefore, the root to shoot ratio of LL was significantly lower than the other two treatments. In addition, the leaf area of lettuce was the largest under LL, and its specific leaf weight was significantly lower than the other two treatments.

### 3.2. Effect of Light Intensities at Night Time on AsA Content of Lettuce under Red and Blue Continuous Light

**Figure 1:**
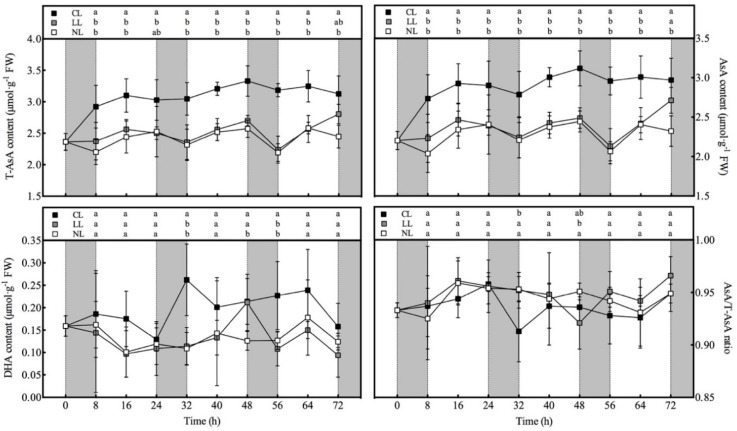
Effects of light intensities at night time on AsA, DHA, and T-AsA contents as well as the ratio of AsA/T-AsA in lettuce leaves under red and blue continuous light. Note: The error line in the figure is the standard deviation. Different letters in the same column indicate significant differences at the *p* < 0.05 level according to Tukey test. (the same below).

It can be seen from the Figure 1 that within 8 h after CL treatment (at the end of the first dark period), the contents of T-AsA and AsA increased significantly. The T-AsA content of lettuce under CL increased by 23.0% and 32.8% compared with LL and NL, respectively. Compared with the LL and NL, AsA content of lettuce increased under CL by 22.5% and 34.2%, respectively. During the period of 8–56 h, the contents of T-AsA and AsA under LL and NL showed consistent diurnal changes, increasing gradually in the light period and decreasing gradually in the dark period. There was no significant difference in T-AsA and ASA contents between the two treatments. The DHA content under CL was the highest within 72 h, but the difference from other treatments reached a significant level only at 32, 48, and 56 h, and the DHA content of the three treatments did not show a diurnal variation.

### 3.3. Effect of Light Intensities at Night Time on AsA Synthase Activity of Lettuce under Red and Blue Continuous Light

**Figure 2:**
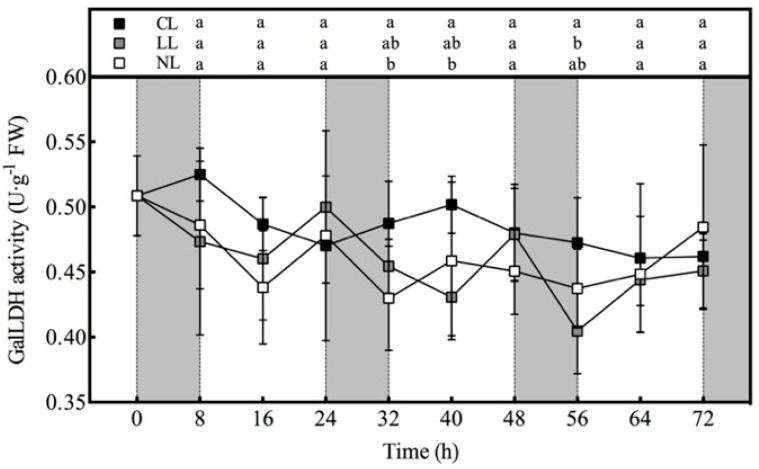
Effects of light intensities at night time on the activity of GalLDH in lettuce leaves under red and blue continuous light.

It can be seen from the Figure 2 that the GalLDH activities of the three treatments did not present a very regular rhythmic characteristic like the AsA content. The GalLDH activities of NL and LL showed a downward trend in the dark period. During the whole period, except for 24 and 72 h, GalLDH activity was the highest under CL. Among them, the GalLDH activity under CL was significantly higher than NL at 32 and 40 h, and was significantly higher than LL at 56 h. There was no significant difference in the GalLDH activities of NL and LL during the whole experiment.

### 3.4. Effect of Light Intensities at Night Time on AsA-GSH Cycle Related Enzyme Activity of Lettuce under Red and Blue Continuous Light

**Figure 3:**
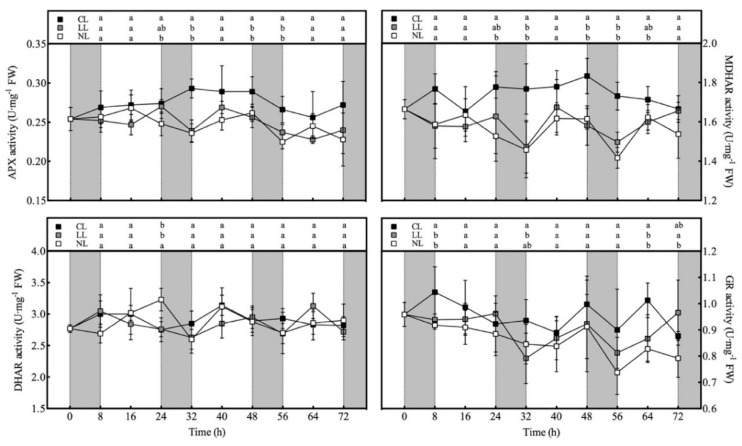
Effects of light intensities at night time on the activities of APX, MDHAR, DHAR, and GR in lettuce leaves under red and blue continuous light.

Overall, the activities of APX, MDHAR, and GR of lettuce under CL were the highest among the three treatments except for a few time points, and the differences from the other two treatments reached significant levels at multiple time points (Figure 3). There was no significant difference in the DHAR activities of different light treatments except for 24 h. The DHAR activity of CL was significantly lower than that of NL at 24 h. There was almost no significant difference in the activities of these four enzymes between NL and LL. These four enzyme activities did not show regular diurnal changes like the AsA content, but in the dark period except 0–8 h, the APX, MDHAR, DHAR, and GR activities of NL and LL all showed the same downtrend. Therefore, starting from 8 h, the ratio of APX, MDHAR, and GR activities increase in CL to NL at the end of each dark period was the highest within 24 h.

### 3.5. Effect of Light Intensities at Night Time on H_2_O_2_ content of Lettuce under Red and Blue Continuous Light

**Figure 4:**
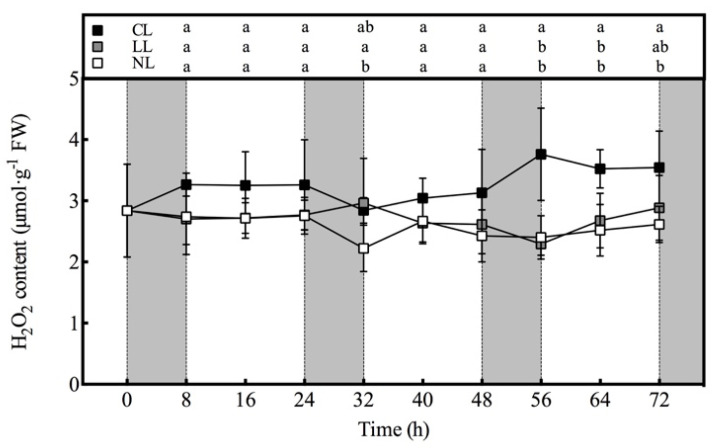
Effects of light intensities at night time on the H_2_O_2_ content in lettuce leaves under red and blue continuous light.

It can be seen from the Figure 4 that, except for 32 h, the H_2_O_2_ content in the lettuce under CL during the entire experiment was the highest among the three treatments. However, in the first 48 h, the H_2_O_2_ content under CL was not significantly different from that of NL, while the H_2_O_2_ content under CL increased significantly within 48–56 h, and subsequently maintained at a significantly higher level than NL. The H_2_O_2_ content of LL was almost the same as that of NL, and was significantly higher than that of NL only at 32 h. In general, the H_2_O_2_ contents of the three treatments did not show regular diurnal changes.

### 3.6. Effect of Light Intensities at Night Time on Fv/Fm value of Lettuce under Red and Blue Continuous Light

**Figure 5:**
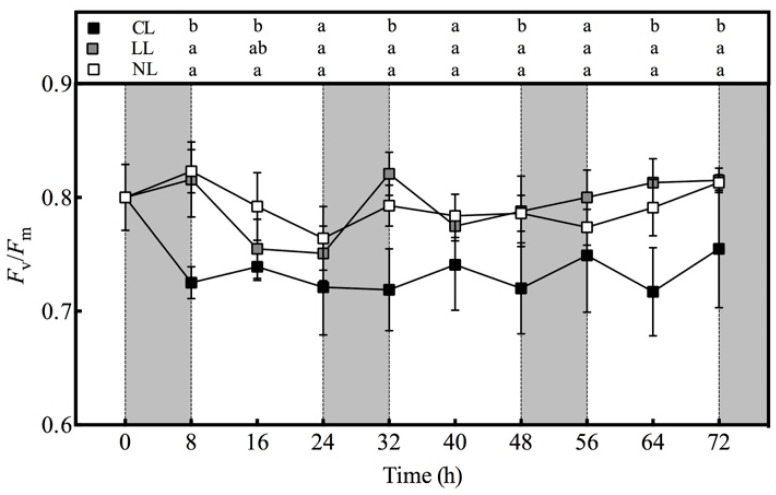
Effects of light intensities at night time on the Fv/Fm value of lettuce leaves under red and blue continuous light.

Compared with the NL, the CL caused a sharp decrease in the Fv/Fm of lettuce within 0–8 h after treatment, from 0.80 to 0.73, and then almost maintained in the range of 0.72–0.75 (Figure 5). During the whole experiment, except for 24, 40, and 56 h, the difference in Fv/Fm between CL and NL reached a significant level. Unlike CL, LL had no significant effect on Fv/Fm. The change trend of Fv/Fm value with time under LL was almost the same as NL. On the whole, the Fv/Fm values of the three treatments did not show regular rhythm characteristics.

## 4. Discussion

Under natural light conditions in greenhouses, the lettuce biomass can be significantly increased by supplementing light to continuous light in the dark period. Under artificial light conditions, continuous light with constant light intensity has also been proved to promote the growth of lettuce [39]. The results of this study showed that compared with NL, CL significantly increased the above-ground and root biomass (fresh weight and dry weight), dry matter content, and specific leaf weight of lettuce, but had no significant effect on leaf area. These research results were consistent with our previous research results, but due to the different growth days, the increase ratio of each index of lettuce under CL was different [40]. Combined with the results of our previous studies, it could be confirmed that continuous light with normal growth light intensity (200 μmol·m^−2^·s^−1^) within a certain period of time (at least 15 d) could promote the growth of lettuce biomass by increasing specific leaf weight instead of leaf area. However, at the same time, the water retention capacity of the stomata may be reduced, which would aggravate the loss of water in the lettuce and caused the moisture content to decrease [41]. In addition, compared with the NL, LL only slightly increased the above-ground biomass of lettuce. Relatively speaking, the root biomass decreased by a greater proportion under LL, making the root-to-shoot ratio significantly lower than NL. This showed that the continuous light of low light (20 μmol·m^−2^·s^−1^) in the dark period was not enough to promote the accumulation of dry matter above the ground, but it could inhibit the transfer of photosynthetic products to the root system.

In our previous research, we measured the dynamic changes of AsA content in lettuce within 3–12 days under CL, and found that the increase in AsA content of CL mainly occurred within 3 days after treatment [40]. Therefore, on the basis of previous research, we monitored the AsA content changes within 3 d (72 h) under continuous light. The results showed that the AsA content increased significantly in the first dark period (8 h) after treatment, and then remained at a relatively stable level and slightly increased. Previous studies found that when *Arabidopsis thaliana* grown in 16/8 h (light/dark period) was transferred to continuous light with the same light intensity, the AsA content increased rapidly within 24 h and stabilized after 48 h [21]. These results indicated that the AsA content responded quickly to continuous light, and under stable continuous light condition, the AsA content of plants could adapt to continuous light after 24–48 h, and remained relatively stable. At the same time, the results of this study also showed that LL could not promote the increase of AsA content in lettuce, which showed that under continuous light, the increase of AsA content had nothing to do with the continuous light signal, which depended on the light intensity.

The content of AsA is regulated by the metabolic processes of its synthesis, oxidative decomposition, and reduction regeneration. In this study, the response of AsA metabolism-related enzyme activity under CL for 0–3 d was almost the same as the response of that for 3–12 d in our previous experiment. Overall, the activities of GalLDH, APX, MDHAR, and GR under CL were higher than those under NL, and reached significant differences at multiple time points, while DHAR activity did not differ significantly under different treatments. This indicated that the increase of AsA content within 72 h under CL was mainly due to the increase of AsA synthesis and AsA reduction regeneration, which was consistent with the mechanism of CL regulating AsA content within 3–15 days [40]. Under some stress conditions, the increase of AsA was also due to the increased efficiency of MDHAR and DHAR in catalyzing the reduction of MDHA and DHA to AsA [32,42]. APX is a key signal that regulates cellular H_2_O_2_ level [43]. In this study, APX activity had a significant regulatory effect on H_2_O_2_ level. During the 24–48 h period, the H_2_O_2_ level under CL decreased with the increase of APX activity. During the 48–64 h period, the H_2_O_2_ level rose with the decrease of APX activity.

The matching degree of plant biological rhythm with the external environment is very important for plant growth. Arabidopsis mutants with too long or too short biological clocks contained more chlorophyll and biomass when grown under light-dark cycle conditions consistent with the endogenous rhythm [44]. Synchronization of internal and external circadian rhythms could promote vegetative growth and reproductive growth of plant in the most suitable time, and enhanced the adaptability and viability of plants [44,45,46]. Under normal photoperiod, there were also circadian rhythms in the AsA content and AsA metabolism-related enzyme activities in plants [19]. In this study, there were regular diurnal changes in the AsA contents under both NL and LL, and the AsA contents increased during the light period and decreased during the dark period, which was consistent with the results of previous studies. Except for the first 8 h after CL, similar diurnal changes were also observed within 8–72 h, but the daily fluctuation of AsA content was smaller, and the circadian rhythm had a tendency to disappear gradually. One of the characteristics of plant circadian rhythm is that it can maintain the original periodic rhythm under constant temperature and continuous light (or continuous dark conditions). At the same time, when the photoperiod of the external environment changes, the rhythm of the plant will change accordingly, so that the final biological rhythm of the plant is kept synchronized with the new environmental cycle. The changes in the AsA content of each treatment in this study also verified the feature of biological rhythm. In addition, although the enzyme activities in this study did not show the same regular diurnal changes as the AsA content, it could still be observed that the activities of GalLDH, APX, MDHAR, and GR under the two treatments of NL and LL all decreased during the dark period. This showed that the circadian rhythm was dependent on light intensity rather than light signal. In the light period, the trend of changes in these enzyme activities was not consistent, which may be due to two reasons. One was that the time interval between sampling points was too long, and the peaks and valleys of enzyme activity changes were missed. Second, the rhythm characteristics of plants did not completely coincide with the rhythm of the external environment, and there was a certain deviation between them.

The response of Fv/Fm to continuous light was similar to that of AsA content. Its value dropped rapidly within 8 h after CL treatment, and then remained at a relatively stable level. This indicated that the adjustment of AsA content by CL was closely related to the photosynthetic system. Inhibitors of the photosynthetic electron transport chain could inhibit the increase of AsA in *Arabidopsis thaliana* under continuous light, indicating that AsA regulation was closely related to photosynthetic electron transport [21].

## 5. Conclusions

CL promoted the accumulation of lettuce biomass, while LL did not significantly promote the biomass, but reduced the root–shoot ratio. The AsA content was significantly increased by CL, and it increased significantly within 8 h after light treatment and then remained stable. DHAR did not play a role in increasing the AsA content under CL. There was no significant difference in the AsA contents between NL and LL showing the same circadian rhythm characteristics, while the enzyme activities under the two treatments kept a consistent downward trend only during the dark period. The Fv/Fm value under CL also decreased significantly within 8 h after light treatment, and then remained stable, indicating that there was a close relationship between the regulation of AsA by CL treatment and the photosynthetic efficiency of photosystem II. In conclusion, the dynamic responses of ascorbate pool and metabolism enzyme activities in lettuce to light intensity at night time under continuous light indicated that light signals did not participate in the metabolic regulation of AsA.

## Figures and Tables

**Table 1:** Light intensities of light period and dark period for each treatment.

Treatments	Light Period (6:00–22:00)	Dark Period (22:00–6:00)
NL	200 μmol·m^−2^·s^−1^	0 μmol·m^−2^·s^−1^
LL	200 μmol·m^−2^·s^−1^	20 μmol·m^−2^·s^−1^
CL	200 μmol·m^−2^·s^−1^	200 μmol·m^−2^·s^−1^

**Table 2:** Effects of light intensities at night time on lettuce growth under red and blue continuous light.

Treatments	Shoot Fresh Weight (g)	Root Fresh Weight (g)	Shoot Dry Weight (g)	Root Dry Weight (g)	Root/Shoot Ratio	Dry Matter Content (%)	Specific Leaf Weight (g/dm^2^)	Leaf Area (dm^2^)
NL	35.0 ± 2.1b	7.3 ± 0.7ab	1.94 ± 0.13b	0.36 ± 0.02b	0.186 ± 0.010a	5.5 ± 0.25b	3.59 ± 0.05b	6.08 ± 0.65a
LL	37.0 ± 1.9b	6.0 ± 1.0b	2.11 ± 0.11b	0.34 ± 0.03b	0.161 ± 0.007b	5.8 ± 0.15b	3.26 ± 0.16c	6.62 ± 0.47a
CL	43.8 ± 3.7a	7.8 ± 0.8a	2.61 ± 0.33a	0.47 ± 0.06a	0.182 ± 0.006a	6.5 ± 0.28a	3.82 ± 0.05a	6.24 ± 0.97a

Note: The data in the table are the means ± standard deviations. Different letters indicate significant differences between different treatments at *p* < 0.05 according to the Tukey test.

## Data Availability

Data is contained within the article.
